# Haplotypes of *HTRA1* rs1120638, *TIMP3* rs9621532, *VEGFA* rs833068, *CFI* rs10033900, *ERCC6* rs3793784, and *KCTD10* rs56209061 Gene Polymorphisms in Age-Related Macular Degeneration

**DOI:** 10.1155/2019/9602949

**Published:** 2019-09-08

**Authors:** Rasa Liutkeviciene, Alvita Vilkeviciute, Greta Gedvilaite, Kriste Kaikaryte, Loresa Kriauciuniene

**Affiliations:** Neuroscience Institute, Lithuanian University of Health Sciences, Medical Academy, Eiveniu 2, Kaunas LT-50161, Lithuania

## Abstract

**Background:**

To determine the impact of *HTRA1* rs1120638, *TIMP3* rs9621532, *VEGFA* rs833068, *CFI* rs10033900, *ERCC6* rs3793784, and *KCTD10* rs56209061 genotypes on the development of age-related macular degeneration (AMD) in the Lithuanian population.

**Methods:**

A total of 916 subjects were examined: 309 patients with early AMD, 301 patients with exudative AMD, and 306 healthy controls. The genotyping of *HTRA1* rs11200638, *TIMP3* rs9621532, *VEGFA* rs833068, *CFI* rs10033900, *ERCC6* rs3793784, and *KCTD10* rs56209061 was carried out using the RT-PCR method.

**Results:**

Our study showed that single-nucleotide polymorphisms rs3793784 and rs11200638 were associated with increased odds of early and exudative AMD, and the variant in *KCTD10* (rs56209061) was found to be associated with decreased odds of early and exudative AMD development after adjustments for age and gender in early AMD analysis and after adjustments only for age in exudative AMD. The haplotype containing two minor alleles C-A and the G-A haplotype in rs3793784-rs11200638 were statistically significantly associated with an increased risk of exudative AMD development after adjustment for age, while the G-G haplotype showed a protective role against early and exudative AMD and the haplotype C-G in rs3793784-rs11200638 was associated with a decreased risk only of exudative AMD development.

**Conclusions:**

Our study identified two markers, rs11200638 and rs3793784, as risk factors for early and exudative AMD, and one marker, rs56209061, as a protective factor for early and exudative AMD development. The haplotypes constructed of rs3793784-rs11200638 were found to be associated with AMD development, as well.

## 1. Introduction

Age-related macular degeneration (AMD) affects the macula (the central part of the retina) and is the most common cause of visual loss in persons over the age of 60 in developed countries [1]. Population estimates have placed the prevalence of AMD approximately from 7% to 10% in adults aged 40-90 yrs. [2–4]. The 10-year incidence of neovascular AMD is 4.1% in persons older than 75 years [5].

In 2002, there were 13.8% blind people due to AMD according to the data of the Lithuanian Medical Social Expertise Commission [6], and it takes second place after glaucoma [6].

AMD is a progressive eye disease that has been linked with several pathological factors, i.e., chronic oxidative stress, autophagy decline, and inflammation [7–9]. However, the full etiology of AMD remains unclear and the treatment options are limited. AMD is a challenging disease to study from a genetic perspective too, because, unlike disorders that exhibit Mendelian inheritance patterns (where one gene and one mutation within that gene is responsible for the phenotype observed in a given family), the development and severity of complex diseases like AMD are influenced by multiple factors. The high prevalence of the disease implies that there is more than one gene or environmental factor as well as interactions among these factors, which influence an individual's susceptibility to the disease [10].

Early AMD is usually asymptomatic. Early AMD is defined as the presence of drusen and retinal pigmentary abnormalities; late AMD includes dry AMD (geographic atrophy of the retinal pigmentary epithelium in the absence of neovascular AMD) or neovascular AMD (detachment of the retinal pigment epithelium, hemorrhages, and/or scars).

AMD is a disease of multifactorial etiology, the development of which is determined by environmental and genetic risk factors. In addition to these factors, various single-nucleotide polymorphisms (SNPs) have been widely reported to be associated with AMD [11]. Genome-wide association studies (GWAS) have confirmed multiple AMD-associated loci. Recently, a large-scale GWAS with thousands of cases and controls reported several additional AMD-associated loci, including rs9621532 near the tissue inhibitor of metalloproteinase 3 (TIMP3) and the synapsin III (SYN3) region of chromosome 22q12.3 [11]. *HTRA1*, the third gene in the 10q26, is highly conserved among species and has several variants that have consistently been found to be associated with AMD [12–18]. One of the most consistently AMD-associated variants is the SNP rs11200638, located within the putative *HTRA1* promoter [15, 17–21]. As the lipid level has been implicated as a major risk factor in AMD [22, 23], GWAS analyze the associations between lipid-trait genes and AMD risk [24–26]. A recent study has shown that two intronic variants rs11066782 and rs11613718 in *KCTD10* or potassium channel tetramerisation domain-containing 10 gene were associated with high-density lipoprotein cholesterol (HDL-C) concentrations and with coronary heart disease (CHD) risk [27], while rs2338104 in the *KCTD10* gene located in chromosome 12q24 was associated with AMD development [28]. The other intronic variant, rs5620906 in *KCTD10*, was even related to the advanced AMD subtypes [29].

Today, it is known that the complement system is involved in the pathogenesis of AMD [30]. Therefore, different types of studies were performed to detect the association between CFI polymorphisms and AMD. Numerous researches have shown *CFI* gene's role in AMD pathogenesis [31–35]. The *CFI* gene is located on chromosome 4q25 and encodes complement factor I (FI), which is an important component of the complement system. Factor I is composed of one light and one heavy polypeptide chains held together by disulfide bonds. These chains are encoded by the *CFI* gene. The light chain has a serine protease domain. The main function of factor I is to cleave C4b and inactivate C3 [36].

Another gene, the Excision Repair Cross-Complementing Group 6 (*ERCC6*) gene, which is located on human chromosome 10 at q11.23 and has 84,171 bases with 21 exons, may be also associated with AMD. Loss of function mutations in the *ERCC6* cause the autosomal recessive disorder Cockayne syndrome, which includes a lot of severe physical and neurologic peculiarities, with premature aging and retinopathy as signs of the disease. These observations led to the hypothesis that (oxidative) DNA damage and insufficient DNA repair contribute to aging pathology, including AMD [37, 38].

Vascular endothelial growth factor A (*VEGFA*) is a member of the *VEGF*-related polypeptide family*. VEGF* plays a key role in increased vascular permeability, angiogenesis, cell growth, and migration of endothelial cells [39–42]. Therefore, it is believed that the polymorphism of the *VEGFA* gene rs833068 can be associated with AMD. Also, it may influence the visual outcomes in neovascular AMD [43, 44].

There have already been many SNPs analyzed that were known to be involved in lipid metabolism, tissue remodeling, and DNA damage mechanisms ,and their associations with AMD development were revealed [45, 46].

We believe that selected SNPs and previously analyzed genetic molecular marker complexes are necessary for understanding, diagnosing, and forecasting AMD. Genetic biomarkers could be useful as biological markers of AMD diagnosis, progression, prognosis, and maybe treatment with anti-VEGF and could be adapted for practical work. While the association studies of these SNPs have not been conducted in both (early and exudative) AMD forms previously, we decided to involve patients with early and exudative AMD and compare the genetic differences between developments of early and exudative AMD.

Most studies focussing on certain molecular pathways involved in the AMD pathogenesis are done in Asian regions. As we know, allele frequencies are known to vary with latitude in different geographic regions which demands further researches to better understand risk factors of AMD development in the Lithuanian population.

Also, we believe that genetic variations combined with other risk factors or molecular changes can benefit physicians in creating personalized genetic therapies and/or lifestyle programs for increased-risk individuals in the future.

In this study, we limited our study to genes in biological pathways relevant to AMD pathogenesis, including the complement system regulation (*CFI*) [36], extracellular matrix deposition and angiogenesis (*HTRA1*) [12], increased vascular permeability (*VEGFA*) [39], or degradation of the extracellular matrix (*TIMP3*) [11]. We also included SNPs in the gene associated with lipid metabolism (*KCTD10*) [27] and in the *ERCC6* gene associated to DNA damage caused by the oxidative stress and leading to the loss of protein function in aging pathology [38].

Furthermore, allele frequencies are known to vary with latitude depending on the region; for this reason, we aimed to determine associations between the *HTRA1* rs1120638, *TIMP3* rs9621532, *VEGFA* rs833068, *CFI* rs10033900, *ERCC6* rs3793784, and *KCTD10* rs56209061 gene polymorphisms and the development of AMD in the Lithuanian population.

## 2. Materials and Methods

Our study was conducted in the Department of Ophthalmology, Hospital of Lithuanian University of Health Sciences, and in the Laboratory of Ophthalmology, Neuroscience Institute, Lithuanian University of Health Sciences. The study was approved by the Ethics Committee for Biomedical Research, Lithuanian University of Health Sciences (No. BE-2-/13). All subjects provided written informed consent in accordance with the Declaration of Helsinki.

### 2.1. Controls and Patients with Age-Related Macular Degeneration Group Formation

A total of 916 subjects were examined, including patients with early AMD (*n* = 309), patients with exudative AMD (*n* = 301), and healthy controls (*n* = 306) (Table 1).

The control group consisted of subjects who had no ophthalmologic pathology on examination and who agreed to take part in this study. Also, according to the age of AMD patients, only subjects older than 55 years old were involved in the control group to avoid the age effect in our study.

AMD patients were not eligible for enrollment in the study if they had (i) unrelated eye disorders, e.g., high refractive error, cloudy cornea, lens opacity (nuclear, cortical, or posterior subcapsular cataract) except for minor opacities, keratitis, acute or chronic uveitis, glaucoma, or diseases of the optic nerve; (ii) systemic illnesses, e.g., diabetes mellitus, malignant tumors, systemic connective tissue disorders, chronic infectious diseases, or conditions following organ or tissue transplantation; and (iii) ungraded color fundus photographs resulting from obscuration of the ocular optic system or because of fundus photograph quality.

The ophthalmological evaluation, DNA extraction, and genotyping used in our research were described in previous studies [47, 48].

### 2.2. Statistical Analysis

Statistical analysis was performed using the SPSS/W 20.0 software (Statistical Package for the Social Sciences for Windows Inc., Chicago, Illinois, USA). Data are expressed as absolute numbers with percentages. Frequencies of the genotypes are expressed in percentages.

Hardy-Weinberg analysis was performed to compare the observed and expected frequencies of *CFI* rs10033900, *ERCC6* rs3793784, *VEGFA* rs833068, *HTRA1* rs11200638, *TIMP3* rs9621532, and *KCTD10* rs56209061 using the *χ*^2^ test in all groups. The distribution of *CFI* rs10033900, *ERCC6* rs3793784, *VEGFA* rs833068, *HTRA1* rs11200638, *TIMP3* rs9621532, and *KCTD10* rs56209061 in the early and exudative AMD and control groups was compared using the *χ*^2^ test or the Fisher exact test. Binomial logistic regression analysis was performed to estimate the impact of genotypes on early and exudative AMD development. Odds ratios and 95% confidence intervals are presented.

For multiple comparisons of the SNPs studied, we used a significance value corrected by the Bonferroni approach. This adjustment was done for *CFI* rs10033900, *ERCC6* rs3793784, *VEGFA* rs833068, *HTRA1* rs11200638, *TIMP3* rs9621532, and *KCTD10* rs56209061 resulting in a “corrected” significance threshold of *α* = 0.008 (0.05/6, since we analyzed 6 different SNPs) for genetic data.

Haplotype analysis was restricted to polymorphisms located on the same chromosome, the haplotype rs3793784-rs11200638. Estimation of the haplotype frequencies and haplotype association tests for the haplotypes with frequencies of at least 5% was carried out using PLINK software version 1.07 [49]. Linkage disequilibrium (LD) analysis was assessed by *D*′ and *r*^2^ measures.

Differences were considered statistically significant when *P* < 0.008.

## 3. Results

A total of 916 subjects were examined, including patients with early AMD (*n* = 309), patients with exudative AMD (*n* = 301), and healthy controls (*n* = 306) (Table 1).

We performed the Hardy-Weinberg equilibrium (HWE) testing among the control group subjects, and the analysis showed that the distribution of the genotypes of all SNPs in the control group did not deviate from HWE (Table 2).

In this study, we analyzed six SNPs in the early AMD, exudative AMD, and control groups and found statistically significant differences in the genotype distribution of *ERCC6* rs3793784, *HTRA1* rs11200638, and *KCTD10* rs56209061 in early and/or exudative AMD groups compared to the control group (Table 3). Also, it was found that the allele A at rs11200638 was more frequent in early AMD patients than in controls (0.35 vs. 0.28, *P* = 0.005) as well as in the exudative AMD group compared to controls (0.54 vs. 0.28, *P* < 0.001); the A allele at rs562209061 was statistically significantly less frequent in the early AMD group than in the control group (0.07 vs. 0.13, *P* < 0.001) and in the exudative AMD group compared to controls (0.08 vs. 0.13, *P* = 0.006) (Table 3).

Binomial logistic regression was performed to evaluate the impact of SNPs on early and exudative AMD development. It revealed that the genotype CC at rs3793784 was associated with 2-fold increased odds of early AMD development under the recessive model (OR = 2.007; 95% CI: 1.255-3.209; *P* = 0.004) (Table 4). The allele A at rs11200638 was also associated with increased odds of early AMD development under the additive model (OR = 1.387; 95% CI: 1.096-1.755; *P* = 0.007) (Table 4). The rs56209061 polymorphism was associated with decreased odds of early AMD development: the GA genotype revealed decreased odds of early AMD under the codominant (OR = 0.471; 95% CI: 0.307-0.724; *P* = 0.001) and overdominant (OR = 0.478; 95% CI: 0.312-0.733; *P* = 0.001) models and the GA+AA was statistically significantly associated with early AMD under the dominant model (OR = 0.463; 95% CI: 0.305-0.704; *P* < 0.001), as well as each A allele at rs56209061 under the additive model (OR = 0.491; 95% CI: 0.332-0.726; *P* < 0.001) (Table 4).

Binomial logistic regression in the exudative AMD and control groups were adjusted for age because patients with exudative AMD were statistically significantly older than control subjects. The genotype CC at rs3793784 was associated with increased odds of exudative AMD development under the codominant and recessive models (OR = 2.271; 95% CI: 1.333-3.870; *P* = 0.003 and OR = 2.427; 95% CI: 1.483-3.970; *P* < 0.001, respectively). The rs11200638 AA genotype was associated with increased odds of exudative AMD development under the codominant and recessive models (OR = 8.683; 95% CI: 5.090-14.810; *P* < 0.001 and OR = 5.146; 95% CI: 3.174-8.344; *P* < 0.001, respectively), as well as the AA+GA under the dominant model (OR = 3.701; 95% CI: 2.569-5.332; *P* < 0.001) and the GA genotype under the codominant model (OR = 2.603; 95% CI: 1.761-3.846; *P* < 0.001) (Table 5). Also, each A allele at rs11200638 was associated with increased odds of exudative AMD development under the additive model (OR = 2.876; 95% CI: 2.230-3.709; *P* < 0.001) (Table 5). The associations of the rs56209061 polymorphism with exudative AMD development were found under the additive model (OR = 0.583; 95% CI: 0.394-0.863; *P* = 0.007) (Table 5).

Unfortunately, no associations of the *VEGFA* rs833068 were found with early or exudative AMD development in the Lithuanian population.

### 3.1. Haplotype Associations with AMD

In this section, we performed an association analysis between the risk of early and exudative AMD and the haplotypes of rs3793784-rs11200638. The *r*^2^ values were 0 and 0.003 in early AMD and exudative AMD analyses, respectively; for the haplotype block, *D*′ values were 0.008 and 0.056 in early AMD and exudative AMD analyses, respectively, which shows that rs3793784 and rs11200638 are in weak linkage disequilibrium.

The haplotype containing two minor alleles C-A in rs3793784-rs11200638 and the G-A haplotypes were statistically significantly associated with an increased risk of exudative AMD development after adjustment for age (Table 6), while the G-G haplotype showed the protective role against both early and exudative AMD and the haplotype C-G in rs3793784-rs11200638 was associated with a decreased risk only of exudative AMD development (Table 6).

## 4. Discussion

Different genes may be responsible for AMD development, and various AMD clinical subtypes may be found in our practice [10]. In our research, we analyzed the *HTRA1* rs1120638, *TIMP3* rs9621532, *VEGFA* rs833068, *CFI* rs10033900, *ERCC6* rs3793784, and *KCTD10* rs56209061 genotype effects on AMD development in the Lithuanian population.

One of our evaluated genes, *HTRA1*, has been shown to be associated with an increased risk of wet AMD in the Chinese population [50]. Our study is also in agreement with this, and we found that the *HTRA1* rs11200638 minor allele was associated with the increased odds of both early and exudative AMD development. It is known that the *HTRA1* gene encodes a serine protease expressed in RPE and drusen. The protein appears to regulate the degradation of extracellular matrix proteoglycans and works in conjunction with other extracellular matrix degradation enzymes (i.e., collagenases and metalloproteinases). *HTRA1* also binds to and inhibits the transformation of growth factor-beta (TGF-*β*), a factor known to play a crucial role in extracellular matrix deposition and angiogenesis [12, 51]. Therefore, it is possible that *HTRA1* may play a role in the regulation of the Bruch's membrane and the growth of vessels into the RPE. One study has concluded that the *HTRA1* SNP rs11200638 is the most likely causal variant of AMD at the 10q26 locus among the Han Chinese population and estimated the combined population attributable risk for *CFH* and *HTRA1* alleles to be 75% [12]. Recently, rs2284665 in *ARMS2/HTRA1* has also been identified as affecting the growth of CNV in AMD [52]. Overall, variants of *ARMS2/HTRA1* genes confer a major risk of the development of AMD. Tang et al. in meta-analysis have summarized a strong evidence of the association between the *HTRA1* -512G>A polymorphism and AMD and indicated a codominant model of action [53]. The higher levels of *HTRA1* expression in RPE cells have been proven by a group of scientists as well [54]. Most recently, a study has reported a significant association between AMD and a haplotype containing both the *ARMS2* indel and the *HTRA1* promoter rs11200638 variant [18]. This is not surprising given that variants of both *HTRA1* and *ARMS2* are in high linkage disequilibrium [13, 55], and we go in agreement with all these studies talking about the significant role of *HTRA1* in AMD development.

While many other studies have proven the association between *TIMP3* rs9621532 and AMD, we did not reveal any significant results in our population. *TIMP3* is a senescence protein that regulates extracellular matrix remodeling and is highly expressed in RPE [56]. Mutations in *TIMP3* were implicated in Sorsby's fundus dystrophy (SFD), an autosomal dominant disorder featuring accumulation of macular drusen and progression to CNV and retinal degeneration [57–60]. The increased *TIMP3* protein in Bruch's membrane and drusen of AMD eyes has also been correlated with AMD disease pathology [61]. *TIMP3* serves as an extracellular ligand for *VEGFR-2* and thus directly regulates angiogenesis [62]. Most importantly, the *TIMP3* protein increases in intensity only in older individuals [63], and significantly elevated TIMPs were also detected in Bruch's membrane of AMD patients with respect to age-matched controls [61]. The enhanced expression of *TIMP3* could prevent ECM remodeling and contribute to the thickening of Bruch's membrane with lower integrity because of changes in the homeostatic balance of turnover, which is precisely what is observed in AMD [61]. The study done by Ardeljan et al. has characterized its phenotypic influence on AMD using three independent study cohorts: case-control studies from the National Eye Institute Clinical Center (NEI, *n* = 397) and the Age-Related Eye Disease Study (*n* = 523) as well as a nested case-control study from the Blue Mountains Eye Study (BMES, *n* = 852). Their data suggest that rs9621532 carriers have a lower risk of developing nAMD, potentially because of the decreased transcription of *TIMP3*. The authors state that the association is still noteworthy and may actually be indicative of an important functional variant [64]. This group of scientists has found that rs9621532 carriers have a lower risk of developing nAMD [64]; however, in our study of the Lithuanian population, we did not find statistically significant results of the rs9621532 being associated with AMD development, even when we included quite a big group of subjects: 309 patients with early AMD, 301 patients with exudative AMD, and 306 healthy controls.

*KCTD10* is a member of the polymerase delta-interacting protein 1 (*PDIP1*) gene family [65], which consists of 3 members, *PDIP1*, *KCTD10*, and *TNFAIP1* [66–69]. It has been found that *KCTD10* can be induced by TNF-*α* and interact with PCNA and the small subunit (p50) of DNA polymerase *δ* [68]. A promoter analysis has shown that *KCTD10* can be regulated by transcription factors [69]. There has even been a novel transcription factor ETV1 identified, which is unique to gastrointestinal stromal tumors (GISTs), suggesting that *KCTD10* functions as a tumor suppressor protein [70]. While the exact functions of *KCTD10* in mammalian development are not clear, the studies have revealed that *KCTD10* is highly expressed in the human heart, skeletal muscle, and placenta, with evidence that *KCTD10* may play a role in the development of the neuroepithelium of the neural tube and dorsal root ganglion [68]. *KCTD10* can also be associated with DNA synthesis and cell proliferation [71], suggesting that this protein may play an important role in tissue development [67, 69]. Also, a recent study has shown that the minor alleles at rs11066782 and rs11613718 in *KCTD10* were associated with higher HDL-C concentrations and a lower CHD risk in the Chinese population [26]. A meta-analysis has been performed to reveal the impact of *KCTD10* rs2338104 on AMD development, and results showed a statistical significance only in African Americans and Mexican Americans (*P* < 0.05) [27]. In another meta-analysis, the distribution of an effective allele at rs56209061 was compared between GA vs. CNV and showed the relation to advanced AMD subtypes [28]. Unfortunately, these associations did not survive corrections for multiple testing [27, 28]. In our study, we revealed opposite results; when comparing with other scientists' groups [27, 28], we found statistically significant associations of the rs56209061 with early and exudative AMD development, considering minor allele A at rs56209061 as a protective marker for early and exudative AMD.

The *CFI* gene spans 63 kb and is composed of 13 exons, of which the first 8 are responsible for the heavy chain and the last 5 for the light chain encoding. The light chain contains the serine protease domain [35]. The *CFI* gene's function is to encode the complement factor I. This factor plays a major role in the complement system. It is expressed by hepatocytes, macrophages, lymphocytes, endothelial cells, and fibroblasts. The complement factor I serum protease plays a major role in the complement system as a C3b and C4b inactivator. Under the presence of cofactors H and C4-binding protein, the complement factor I regulates the complement cascade activation via cleaving the alpha-chains of complement components C4b and C3b. This way, it prevents the assembly of C3 and C5 convertase enzymes [72]. The complement system and *CFI* have been connected in the pathogenesis of AMD. First, studies that have shown an association between *CFI* SNPs and AMD were described by Fagerness et al. A case-control association study for advanced AMD has been performed to identify new regions of the gene of interest. As a result, this research has demonstrated the *CFI* rs10033900 association with AMD in a Caucasian cohort [30]. Other studies have confirmed this connection and have also shown the *CFI* rs10033900 gene polymorphism correlation with AMD in different cohorts [30–34]. For example, Ennis et al. have shown the *CFI* region and AMD susceptibility in a UK cohort [31]. Kondo et al. have indicated the association of the rs10033900 gene polymorphism with the C allele in a Japanese cohort [32]. There have been publications of the link between *CFI* rs10033900 and AMD in two independent cohorts from England and Scotland and also in the Han Chinese population [33, 34]. In our study, we did not determine any associations between *CFI* rs10033900 and AMD development in the Lithuanian population, and the results are in conflict with those of other studies.

We identified the homozygous carriers of the allele C of *ERCC6* rs3793784 as a risk factor for early and exudative AMD, and we are in agreement with other studies [32, 40, 49]. Another study [32] has shown that *ERCC6* may be functionally implicated in the etiology of AMD. They reported that AMD-affected donor eyes had a 50% lower *ERCC6* expression than healthy donor eyes (*P* = 0.018) [32]. A number of studies have also elucidated that a single-nucleotide polymorphism (SNP) in the *ERCC6* promoter regulatory region (rs3793784) was moderately associated with AMD [40, 73]. Combining our data with those of the literature, we hypothesized that the AMD-related reduced transcriptional activity of *ERCC6* expression may be caused by diverse, small, and heterogeneous genetic and/or environmental determinants.

Unfortunately, we found no associations between our last evaluated variant in the *VEGFA* gene (rs833068) and early or exudative AMD development in the Lithuanian population. On the other hand, *VEGFA* has been reported as a predisposing gene to AMD. A recent meta-analysis investigating the genetic susceptibility of AMD has demonstrated that *VEGFA* rs943080 is in a strong linkage disequilibrium (LD) with rs4711751 (*r*^2^ = 1.0 in 1000 Genomes CEU data), which is significantly associated with advanced AMD [43]. There was nominal evidence found of a replication in the same direction as the initial study for *VEGFA* rs943080 (*P* < 0.05) [74]. While some studies found significant *VEGF* impact on AMD development [75–80], others showed opposite results [81]. For example, a study from Spain proved that *VEGFA* or *VEGFR* genes are not associated with the different AMD subtypes. A group of scientists states that further studies in different populations, and with a larger cohort of patients, are needed to confirm these results [81]. Oszajca et al. conducted a case-control study of 200 Polish patients with recognized AMD (dry and wet) who were compared to 100 healthy controls, and they proved that the CC genotype for *VEGF* +936C/T markedly increases the risk of age-related macular degeneration but does not influence its progression [75]. Also, a Chinese study confirmed the importance of *VEGF* on AMD [76]. An Indian study found associations with neovascular AMD for an SNP near *VEGFA* (rs4711751) (OR = 0.64, 95% CI 0.54 to 0.77, *P* = 10^−3^) [77]. An Italian study involved a cohort of 1976 subjects, including 976 patients affected with exudative AMD and 1000 control subjects. This analysis revealed that *VEGFA* was significantly associated with AMD susceptibility. The multivariate analysis showed that age, smoking, dietary habits, and sex, together with the genetic variants, were significantly associated with AMD in our population [78]. A review also described that *VEGFA* was significantly associated with the risk of AMD in the Italian cohort [79]. Finally, a genome-wide single variant analysis of 16,144 advanced AMD patients and 17,832 controls of European ancestry was performed, and it found that the *VEGFA* rs943080 T/C genotype was highly associated with AMD (*P* = 1.1 × 10^−14^**)** [80].

Different genotype distributions can be explained by controversial results in different populations. One genotype may be protective in one population, while the same genotype may increase a likelihood of disease development in another population. Wang et al. confirmed that some gene polymorphisms might be significant in the East Asian population but not in the European population [81]. Gaedigk et al. also confirms the fact that allele frequencies also differ in different populations: in an Ashkenazi Jewish and European cohort, a *CYP2D6*∗*4* allele is the highest, while in an East Asian cohort, it is the lowest [82].

AMD is a disease of multifactorial etiology. Its development is determined not only by genetic but also by modified and unmodified environmental risk factors. Overall, the most important pathogenetic mechanisms causing the development of AMD are the formation of drusen, local inflammation, and neovascularization. But it is still not clear why in some patients early AMD may remain early AMD for all life without any or with very insignificant progression, while in others, it may progress to late AMD (exudative or atrophic forms). The rs3793784 and rs11200638 SNPs and the haplotypes constructed from these two SNPs could be used as biomarkers for the progression of early AMD towards the exudative AMD form, because there were associations found between both early and exudative AMD forms and rs3793784 and rs11200638; however, it is necessary to follow our patients to assess the development and/or progression of AMD. Also, functional studies should be performed to confirm SNP variants as biomarkers for any diagnosis.

## 5. Conclusions

Our study identified two markers (*HTRA1* rs11200638 and *ERCC6* rs3793784) as risk factors for early and exudative AMD and one marker (*KCTD10* rs56209061) as a protective factor for early and exudative AMD development.

The haplotypes constructed of rs3793784-rs11200638 were found to be associated with AMD development, as well.

### 5.1. Strengths and Limitations

Some limitations of this study have to be acknowledged: first, we did not perform functional studies to confirm the associations between SNPs and AMD development; second, the sample size of control subjects was relatively small; any other environmental risk factors were not included in our study.

The strengths of our study include a thorough clinical examination of the patients: all the patients were consulted by a general practitioner, and patients with malignant tumors, rheumatoid diseases, and end-stage liver or renal diseases and other systemic infectious and noninfectious diseases were excluded from the study as well.

## Figures and Tables

**Table 1:** Demographic characteristics.

Characteristic	Group			*P* value
	Early AMD (*n* = 309)	Exudative AMD (*n* = 301)	Control (*n* = 306)	
Gender				
Male (*n*, %)	92 (29.8)	106 (35.2)	95 (31.0)	0.732^∗^
Female (*n*, %)	217 (70.2)	195 (64.8)	211 (69.0)	0.275^∗∗^
Age (years); median (IQR)	73 (12)	76 (11)	71 (15)	0.105^∗^
				<0.001^∗∗^

^∗^Early AMD vs. the control group. ^∗∗^Exudative AMD vs. the control group.

**Table 2:** Analysis of Hardy-Weinberg equilibrium in control group subjects.

SNP	Allele frequencies		Genotype distribution	*P* value
rs10033900	G (0.48)	C (0.52)	73/148/85	0.585
rs3793784	C (0.36)	G (0.64)	38/158/117	0.166
rs833068	A (0.25)	G (0.75)	17/119/170	0.517
rs11200638	A (0.28)	G (0.72)	26/119/161	0.549
rs9621532	C (0.06)	A (0.94)	1/36/269	0.859
rs56209061	A (0.13)	G (0.87)	5/71/230	0.858

**Table 3:** Genotype and allele frequencies of *CFI* rs10033900, *ERCC6* rs3793784, *VEGFA* rs833068, *HTRA1* rs11200638, *TIMP3* rs9621532, and *KCTD10* rs56209061 SNPs in early and exudative AMD and control groups.

Gene/marker	Genotype/allele	Early AMD (*n*, %)	Control group (*n*, %)	*P* value	Exudative AMD (*n*, %)	Control group (*n*, %)	*P* value
*CFI* rs10033900	CC	64 (20.7)	85 (27.8)		83 (27.6)	85 (27.8)	
	CG	166 (53.7)	148 (48.4)	0.122	151 (50.2)	148 (48.4)	0.874
	GG	79 (25.6)	73 (23.9)		67 (22.3)	73 (23.9)	
	C	0.48	0.52	0.124	0.53	0.52	0.808
	G	0.52	0.48		0.47	0.48	

*ERCC6* rs3793784	GG	124 (40.1)	117 (38.2)		111 (36.9)	117 (38.2)	
	GC	128 (41.4)	158 (51.6)	**0.004**	131 (43.5)	158 (51.6)	**0.003**
	CC	57 (18.4)	31 (10.1)		59 (19.6)	31 (10.1)	
	G	0.61	0.64	0.245	0.59	0.64	0.053
	C	0.39	0.36		0.41	0.36	

*VEGFA* rs833068	GG	191 (61.8)	170 (55.6)		185 (61.5)	170 (55.6)	
	GA	98 (31.7)	119 (38.9)	0.175	93 (30.9)	119 (38.9)	0.096
	AA	20 (6.5)	17 (5.6)		23 (7.6)	17 (5.6)	
	G	0.78	0.75	0.271	0.77	0.75	0.436
	A	0.22	0.25		0.23	0.25	

*HTRA1* rs11200638	GG	135 (43.7)	161 (52.6)		70 (23.3)	161 (52.6)	
	GA	129 (41.7)	119 (38.9)	0.021	134 (44.5)	119 (38.9)	**<0.001**
	AA	45 (14.6)	26 (8.5)		97 (32.2)	26 (8.5)	
	G	0.65	0.72	**0.005**	0.46	0.72	**<0.001**
	A	0.35	0.28		0.54	0.28	

*TIMP3* rs9621532	AA	263 (85.1)	269 (87.9)		260 (86.4)	269 (87.9)	
	AC	41 (13.3)	36 (11.8)	0.218	40 (13.3)	36 (11.8)	0.851
	CC	5 (1.6)	1 (0.3)		1 (0.3)	1 (0.3)	
	A	0.92	0.94	0.266	0.93	0.94	0.590
	C	0.08	0.06		0.07	0.06	

*KCTD10* rs56209061	GG	268 (86.7)	230 (75.2)		253 (84.1)	230 (75.2)	
	GA	39 (12.6)	71 (23.2)	**0.001**	46 (15.3)	71 (23.2)	0.021
	AA	2 (0.6)	5 (1.6)		2 (0.7)	5 (1.6)	
	G	0.93	0.87	**<0.001**	0.92	0.87	**0.006**
	A	0.07	0.13		0.08	0.13	

AMD: age-related macular degeneration; *P* values < 0.008 indicated in bold are statistically significant.

**Table 4:** Binomial logistic regression analysis of the *CFI* rs10033900, *ERCC6* rs3793784, *VEGFA* rs833068, *HTRA1* rs11200638, *TIMP3* rs9621532, and *KCTD10* rs56209061 SNPs in early AMD and control groups.

Early AMD			
SNP	Model/genotype/allele	OR; 95% CI; *P*^∗^	AIC
	*Codominant*		
rs56209061	GA vs. GG	0.471; 0.307-0.724; **0.001**	842.895
	AA vs. GG	0.343; 0.066-1.786; 0.204	

	*Dominant*		
rs56209061	GA+AA vs. GG	0.463; 0.305-0.704; <**0.001**	841.036

	*Recessive*		
rs3793784	CC vs. GC+GG	2.007; 1.255-3.209; **0.004**	845.769

	*Overdominant*		
rs56209061	GA vs. GG+AA	0.478; 0.312-0.733; **0.001**	842.698

	*Additive*		
rs11200638	A	1.387; 1.096-1.755; **0.007**	847.056
rs56209061	A	0.491; 0.332-0.726; <**0.001**	841.104

OR: odds ratio; 95% CI: 95% confidence interval; *P* values < 0.008 indicated in bold are statistically significant; AIC: Akaike information criterion.

**Table 5:** Binomial logistic regression analysis of the *CFI* rs10033900, *ERCC6* rs3793784, *VEGFA* rs833068, *HTRA1* rs11200638, *TIMP3* rs9621532, and *KCTD10* rs56209061 SNPs in the exudative AMD and control groups.

Exudative AMD			
SNP	Model/genotype/allele	OR; 95% CI; *P*^∗^	AIC
	*Codominant*		
rs3793784	GC vs. GG	0.889; 0.618-1.279; 0.526	780.990
	CC vs. GG	2.271; 1.333-3.870; **0.003**	

rs11200638	GA vs. GG	2.603; 1.761-3.846; **<0.001**	718.588
	AA vs. GG	8.683; 5.090-14.810; **<0.001**	

	*Dominant*		
rs11200638	GA+AA vs. GG	3.701; 2.569-5.332; <**0.001**	739.869

	*Recessive*		
rs3793784	CC vs. GC+GG	2.427; 1.483-3.970; <**0.001**	779.392
rs11200638	AA vs. GA+GG	5.146; 3.174-8.344; <**0.001**	740.394

	*Additive*		
rs11200638	A	2.876; 2.230-3.709; <**0.001**	716.992
rs56209061	A	0.583; 0.394-0.863; **0.007**	785.038

∗*P* was adjusted for age in logistic regression models. OR: odds ratio; 95% CI: 95% confidence interval; *P* values < 0.008 indicated in bold are statistically significant; AIC: Akaike information criterion.

**Table 6:** Haplotypes associated with AMD.

Single-nucleotide polymorphisms	Haplotype	Frequency (cases)	Frequency (controls)	Chi square	Degree of freedom	*P* value^∗^
Early AMD						
rs3793784-rs11200638	C-A	0.1343	0.1014	9.554	1	0.051
	G-A	0.2196	0.1771	3.485	1	0.054
	C-G	0.2569	0.2586	0.004	1	0.943
	G-G	0.3892	0.4629	6.840	1	**0.007**

Exudative AMD						
rs3793784-rs11200638	C-A	0.2371	0.1069	36.30	1	**<0.001**
	G-A	0.3077	0.1716	30.97	1	**<0.001**
	C-G	0.1765	0.2530	10.55	1	**<0.001**
	G-G	0.2787	0.4685	46.75	1	**<0.001**

∗*P* value adjusted for age in exudative AMD analysis; *P* values < 0.008 indicated in bold are statistically significant.

## Data Availability

Data will be provided in case a request is made by editors, reviewers, or scientists.

## Abbreviations

AMD: Age-related macular degeneration
SNPs: Single-nucleotide polymorphisms
RPE: Retinal pigment epithelium
BrM: Bruch's membrane
GA: Geographic atrophy.

## References

[1] Klein R., Peto T., Bird A., Vannewkirk M. R. (2004). The epidemiology of age-related macular degeneration. *American Journal of Ophthalmology*.

[2] Friedman D. S., O’Colmain B. J., Munoz B. (2004). Prevalence of age-related macular degeneration in the United States. *Archives of Ophthalmology*.

[3] Duan Y., Mo J., Klein R. (2007). Age-related macular degeneration is associated with incident myocardial infarction among elderly Americans. *Ophthalmology*.

[4] Cimbalas A., Paunksnis A., Černiauskienė L. R., Domarkienė S. (2004). Prevalence and risk factors of age-related macular degeneration. *Medicina*.

[5] Klein R., Klein B. E. K., Tomany S. C., Meuer S. M., Huang G.-H. (2002). Ten-year incidence and progression of age-related maculopathy: the Beaver Dam eye study. *Ophthalmology*.

[6] Neverauskiene J., Chaleckiene G., Baniuliene D., Kalasauskiene A. (2003). Blindness incidence in Lithuania. *International Journal of Ophthalmology*.

[7] Mitter S. K., Song C., Qi X. (2014). Dysregulated autophagy in the RPE is associated with increased susceptibility to oxidative stress and AMD. *Autophagy*.

[8] Piippo N., Korkmaz A., Hytti M. (2014). Decline in cellular clearance systems induces inflammasome signaling in human ARPE-19 cells. *Biochimica et Biophysica Acta (BBA) - Molecular Cell Research*.

[9] Ferrington D. A., Sinha D., Kaarniranta K. (2016). Defects in retinal pigment epithelial cell proteolysis and the pathology associated with age-related macular degeneration. *Progress in Retinal and Eye Research*.

[10] DeAngelis M. M., Silveira A. C., Carr E. A., Kim I. K. (2011). Genetics of Age-Related Macular Degeneration: Current Concepts, Future Directions. *Seminars in Ophthalmology*.

[11] Ding X., Patel M., Chan C. C. (2009). Molecular pathology of age-related macular degeneration. *Progress in Retinal and Eye Research*.

[12] Yang Z., Camp N. J., Sun H. (2006). A variant of the HTRA1 gene increases susceptibility to age-related macular degeneration. *Science*.

[13] DeAngelis M. M., Ji F., Adams S. (2008). Alleles in the HtrA serine peptidase 1 gene alter the risk of neovascular age-related macular degeneration. *Ophthalmology*.

[14] Gibbs D., Yang Z., Constantine R. (2008). Further mapping of 10q26 supports strong association of HTRA1 polymorphisms with age-related macular degeneration. *Vision Research*.

[15] Hadley D., Orlin A., Brown G. (2010). Analysis of six genetic risk factors highly associated with AMD in the region surrounding ARMS2 and HTRA1 on chromosome 10, region q26. *Investigative Ophthalmology & Visual Science*.

[16] Mori K., Horie-Inoue K., Gehlbach P. L. (2010). Phenotype and genotype characteristics of age-related macular degeneration in a Japanese population. *Ophthalmology*.

[17] Liang X. Y., Chen L. J., Ng T. K. (2014). FPR1 interacts with CFH, HTRA1 and smoking in exudative age-related macular degeneration and polypoidal choroidal vasculopathy. *Eye (London, England)*.

[18] Yang Z., Tong Z., Chen Y. (2010). Genetic and functional dissection of HTRA1 and LOC387715 in age-related macular degeneration. *PLoS Genetics*.

[19] Jiang H., Qu Y., Dang G. (2009). Analyses of single nucleotide polymorphisms and haplotype linkage of LOC387715 and the HTRA1 gene in exudative age-related macular degeneration in a Chinese cohort. *Retina*.

[20] Bergeron-Sawitzke J., Gold B., Olsh A. (2009). Multilocus analysis of age-related macular degeneration. *European Journal of Human Genetics*.

[21] Farwick A., Dasch B., Weber B. H. F., Pauleikhoff D., Stoll M., Hense H. W. (2009). Variations in five genes and the severity of age-related macular degeneration: results from the Muenster aging and retina study. *Eye (London, England)*.

[22] Reynolds R., Rosner B., Seddon J. M. (2010). Serum lipid biomarkers and hepatic lipase gene associations with age-related macular degeneration. *Ophthalmology*.

[23] Van Leeuwen R., Klaver C. C. W., Vingerling J. R. (2004). Cholesterol and age-related macular degeneration: is there a link?. *American Journal of Ophthalmology*.

[24] Chen W., Stambolian D., Edwards A. O. (2010). Genetic variants near TIMP3 and high-density lipoprotein-associated loci influence susceptibility to age-related macular degeneration. *Proceedings of the National Academy of Sciences of the United States of America*.

[25] Neale B. M., Fagerness J., Reynolds R. (2010). Genome-wide association study of advanced age-related macular degeneration identifies a role of the hepatic lipase gene (LIPC). *Proceedings of the National Academy of Sciences of the United States of America*.

[26] Colak E., Kosanović-Jaković N., Zorić L., Radosavljevic A., Stankovic S., Majkic-Singh N. (2011). The association of lipoprotein parameters and C-reactive protein in patients with age-related macular degeneration. *Ophthalmic Research*.

[27] Sun J., Qian Y., Jiang Y. (2016). Association of KCTD10, MVK, and MMAB polymorphisms with dyslipidemia and coronary heart disease in Han Chinese population. *Lipids in Health and Disease*.

[28] Restrepo N. A., Spencer K. L., Goodloe R. (2014). Genetic determinants of age-related macular degeneration in diverse populations from the PAGE study. *Investigative Ophthalmology & Visual Science*.

[29] Sobrin L., Ripke S., Yu Y. (2012). Heritability and genome-wide association study to assess genetic differences between advanced age-related macular degeneration subtypes. *Ophthalmology*.

[30] Johnson L. V., Leitner W. P., Staples M. K., Anderson D. H. (2001). Complement activation and inflammatory processes in drusen formation and age related macular degeneration. *Experimental Eye Research*.

[31] Fagerness J. A., Maller J. B., Neale B. M., Reynolds R. C., Daly M. J., Seddon J. M. (2009). Variation near complement factor I is associated with risk of advanced AMD. *European Journal of Human Genetics*.

[32] Ennis S., Gibson J., Cree A. J., Collins A., Lotery A. J. (2010). Support for the involvement of complement factor I in age-related macular degeneration. *European Journal of Human Genetics*.

[33] Kondo N., Bessho H., Honda S., Negi A. (2010). Additional evidence to support the role of a common variant near the complement factor I gene in susceptibility to age-related macular degeneration. *European Journal of Human Genetics*.

[34] Cipriani V., Matharu B. K., Khan J. C. (2012). No evidence of association between complement factor I genetic variant rs10033900 and age-related macular degeneration. *European Journal of Human Genetics*.

[35] Qian D., Kan M., Weng X. (2014). Common variant rs10033900 near the complement factor I gene is associated with age-related macular degeneration risk in Han Chinese population. *European Journal of Human Genetics*.

[36] Vyse T. J., Bates G. P., Walport M. J., Morley B. J. (1994). The Organization of the Human Complement Factor I Gene (IF): A Member of the Serine Protease Gene Family. *Genomics*.

[37] Baas D. C., Despriet D. D., Gorgels T. G. M. F. (2010). The ERCC6 gene and age-related macular degeneration. *PLoS One*.

[38] Mousavi M., Armstrong R. A. (2013). Genetic risk factors and age-related macular degeneration (AMD). *Journal of Optometry*.

[39] Gupta S., Johnson S. H., Vasmatzis G. (2017). TFEB-VEGFA (6p21. 1) co-amplified renal cell carcinoma: a distinct entity with potential implications for clinical management. *Modern Pathology*.

[40] Zhao L., Grob S., Avery R. (2013). Common variant in VEGFA and response to anti-VEGF therapy for neovascular age-related macular degeneration. *Current Molecular Medicine*.

[41] Claesson-Welsh L., Welsh M. (2013). VEGFA and tumour angiogenesis. *Journal of Internal Medicine*.

[42] Kokki E., Karttunen T., Olsson V., Kinnunen K., Ylä-Herttuala S. (2018). Human vascular endothelial growth factor A165 expression induces the mouse model of neovascular age-related macular degeneration. *Genes*.

[43] Lorés-Motta L., de Jong E. K., den Hollander A. I. (2018). Exploring the use of molecular biomarkers for precision medicine in age-related macular degeneration. *Molecular Diagnosis & Therapy*.

[44] Abedi F., Wickremasinghe S., Richardson A. J. (2013). Variants in the VEGFA gene and treatment outcome after anti-VEGF treatment for neovascular age-related macular degeneration. *Ophthalmology*.

[45] Black J. R. M., Clark S. J. (2016). Age-related macular degeneration: genome-wide association studies to translation. *Genetics in Medicine*.

[46] Vilkeviciute A., Kriauciuniene L., Chaleckis R., Deltuva V. P., Liutkeviciene R. (2019). RAD51B (rs8017304 and rs2588809), TRIB1 (rs6987702, rs4351379, and rs4351376), COL8A1 (rs13095226), and COL10A1 (rs1064583) gene variants with predisposition to age-related macular degeneration. *Disease Markers*.

[47] Liutkeviciene R., Vilkeviciute A., Kriauciuniene L., Deltuva V. P. (2019). SIRT1 rs12778366, FGFR2 rs2981582, STAT3 rs744166, LIPC rs10468017, rs493258 and LPL rs12678919 genotypes and haplotype evaluation in patients with age-related macular degeneration. *Gene*.

[48] Liutkeviciene R., Vilkeviciute A., Streleckiene G., Kriauciuniene L., Chaleckis R., Deltuva V. P. (2017). Associations of cholesteryl ester transfer protein (CETP) gene variants with predisposition to age-related macular degeneration. *Gene*.

[49] Purcell S., Neale B., Todd-Brown K. (2007). PLINK: a tool set for whole-genome association and population-based linkage analysis. *American Journal of Human Genetics*.

[50] DeWan A., Liu M., Hartman S. (2006). HTRA1 promoter polymorphism in wet age-related macular degeneration. *Science*.

[51] Zhang Y., Marmorstein L. Y. (2010). Focus on molecules: fibulin-3 (EFEMP1). *Experimental Eye Research*.

[52] Akagi-Kurashige Y., Yamashiro K., Gotoh N. (2015). MMP20 and ARMS2/HTRA1 are associated with neovascular lesion size in age-related macular degeneration. *Ophthalmology*.

[53] Tang N. P., Zhou B., Wang B., Yu R. B. (2009). HTRA1 promoter polymorphism and risk of age-related macular degeneration: a meta-analysis. *Annals of Epidemiology*.

[54] An E., Sen S., Park S. K., Gordish-Dressman H., Hathout Y. (2010). Identification of novel substrates for the serine protease HTRA1 in the human RPE secretome. *Investigative Ophthalmology & Visual Science*.

[55] Fritsche L. G., Loenhardt T., Janssen A. (2008). Age-related macular degeneration is associated with an unstable ARMS2 (LOC387715) mRNA. *Nature Genetics*.

[56] Della N. G., Campochiaro P. A., Zack D. J. (1996). Localization of TIMP-3 mRNA expression to the retinal pigment epithelium. *Investigative Ophthalmology & Visual Science*.

[57] Weber B. H. F., Vogt G., Wolz W., Ives E. J., Ewing C. C. (1994). Sorsby’s fundus dystrophy is genetically linked to chromosome 22q13-qter. *Nature Genetics*.

[58] Weber B. H. F., Vogt G., Pruett R. C., Stöhr H., Felbor U. (1994). Mutations in the tissue inhibitor of metalloproteinases-3 (TIMP3) in patients with Sorsby’s fundus dystrophy. *Nature Genetics*.

[59] Felbor U., Stöhr H., Amann T., Schönherr U., Weber B. H. F. (1995). A novel Ser156Cys mutation in the tissue inhibitor of metalloproteinases-3 (TIMP3) in Sorsby’s fundus dystrophy with unusual clinical features. *Human Molecular Genetics*.

[60] Felbor U., Stohr H., Amann T., Schonherr U., Apfelstedt-Sylla E., Weber B. H. (1996). A second independent Tyr168Cys mutation in the tissue inhibitor of metalloproteinases-3 (TIMP3) in Sorsby’s fundus dystrophy. *Journal of Medical Genetics*.

[61] Kamei M., Hollyfield J. G. (1999). TIMP-3 in Bruch’s membrane: changes during aging and in age-related macular degeneration. *Investigative Ophthalmology & Visual Science*.

[62] Qi J. H., Ebrahem Q., Moore N. (2003). A novel function for tissue inhibitor of metalloproteinases-3 (TIMP3): inhibition of angiogenesis by blockage of VEGF binding to VEGF receptor-2. *Nature Medicine*.

[63] Macgregor A. M., Eberhart C. G., Fraig M., Lu J., Halushka M. K. (2009). Tissue inhibitor of matrix metalloproteinase-3 levels in the extracellular matrix of lung, kidney, and eye increase with age. *The Journal of Histochemistry and Cytochemistry*.

[64] Ardeljan D., Meyerle C. B., Agron E. (2013). Influence of TIMP3/SYN3 polymorphisms on the phenotypic presentation of age-related macular degeneration. *European Journal of Human Genetics*.

[65] He H., Tan C. K., Downey K. M., So A. G. (2001). A tumor necrosis factor alpha- and interleukin 6-inducible protein that interacts with the small subunit of DNA polymerase delta and proliferating cell nuclear antigen. *Proceedings of the National Academy of Sciences of the United States of America*.

[66] Zhou J., Hu X., Xiong X. (2005). Cloning of two rat PDIP1 related genes and their interactions with proliferating cell nuclear antigen. *Journal of Experimental Zoology Part A: Comparative Experimental Biology*.

[67] Zhou J., Ren K., Liu X., Xiong X., Hu X., Zhang J. (2005). A novel PDIP1-related protein, KCTD10, that interacts with proliferating cell nuclear antigen and DNA polymerase *δ*. *Biochimica et Biophysica Acta (BBA) - Gene Structure and Expression*.

[68] Zhou J., Fan C., Zhong Y. (2005). Genomic organization, promoter characterization and roles of Sp1 and AP-2 in the basal transcription of mouse PDIP1 gene. *FEBS Letters*.

[69] Liu R., Zhou A., Ren D. (2009). Transcription factor specificity protein 1 (SP1) and activating protein 2alpha (AP-2alpha) regulate expression of human KCTD10 gene by binding to proximal region of promoter. *The FEBS Journal*.

[70] Kubota D., Yoshida A., Tsuda H. (2013). Gene expression network analysis of ETV1 reveals KCTD10 as a novel prognostic biomarker in gastrointestinal stromal tumor. *PLoS One*.

[71] Wang Y., Zheng Y., Luo F. (2009). KCTD10 interacts with proliferating cell nuclear antigen and its down-regulation could inhibit cell proliferation. *Journal of Cellular Biochemistry*.

[72] Fraczek L. A., Martin B. K. (2010). Transcriptional control of genes for soluble complement cascade regulatory proteins. *Molecular Immunology*.

[73] Tuo J., Cho Y., Chew E., Chan C. C. (2009). A variant in 5-UTR of ERCC6 protects against age-related macular degeneration in European decent. *The FASEB Journal*.

[74] Cheng C. Y., Yamashiro K., Jia Chen L. (2015). New loci and coding variants confer risk for age-related macular degeneration in East Asians. *Nature Communications*.

[75] Oszajca K., Szemraj M., Szemraj J., Jurowski P. (2018). Association analysis of genetic polymorphisms and expression levels of selected genes involved in extracellular matrix turnover and angiogenesis with the risk of age-related macular degeneration. *Ophthalmic Genetics*.

[76] Huang Q., Xiang Y. (2018). Polymorphisms in selected genes and their association with age-related macular degeneration in a Chinese population. *Medical Science Monitor*.

[77] Rajendran A., Dhoble P., Sundaresan P. (2018). Genetic risk factors for late age-related macular degeneration in India. *The British Journal of Ophthalmology*.

[78] Cascella R., Strafella C., Longo G. (2018). Uncovering genetic and non-genetic biomarkers specific for exudative age-related macular degeneration: significant association of twelve variants. *Oncotarget*.

[79] Cascella R., Strafella C., Caputo V. (2018). Towards the application of precision medicine in age-related macular degeneration. *Progress in Retinal and Eye Research*.

[80] Fritsche L. G., Igl W., Bailey J. N. C. (2016). A large genome-wide association study of age-related macular degeneration highlights contributions of rare and common variants. *Nature Genetics*.

[81] Wang J., Xu D., Wu X. (2011). Polymorphisms of matrix metalloproteinases in myocardial infarction: a meta-analysis. *Heart*.

[82] Gaedigk A., Sangkuhl K., Whirl-Carrillo M., Klein T., Leeder J. S. (2017). Prediction of CYP2D6 phenotype from genotype across world populations. *Genetics in Medicine*.

